# Towards elimination of measles and rubella in Italy: Progress and challenges

**DOI:** 10.1371/journal.pone.0226513

**Published:** 2019-12-16

**Authors:** Giovanna Adamo, Valentina Baccolini, Azzurra Massimi, Domenico Barbato, Rosario Cocchiara, Carolina Di Paolo, Annamaria Mele, Sara Cianfanelli, Aurora Angelozzi, Fulvio Castellani, Carla Salerno, Claudia Isonne, Antonino Bella, Antonietta Filia, Martina del Manso, Melissa Baggieri, Loredana Nicoletti, Fabio Magurano, Stefania Iannazzo, Carolina Marzuillo, Paolo Villari

**Affiliations:** 1 Department of Public Health and Infectious Diseases, Sapienza University of Rome, Rome, Italy; 2 National Institute for Infectious Diseases Lazzaro Spallanzani IRCCS, Rome, Italy; 3 Department of Infectious Diseases, National Institute of Health, Rome, Italy; 4 National Reference Laboratory for Measles and Rubella, National Institute of Health, Rome, Italy; 5 Infectious Diseases and International Prophylaxis Office, Ministry of Health, Rome, Italy; University of Campania, ITALY

## Abstract

**Introduction:**

In the WHO European Region, endemic transmission of measles and rubella had been interrupted by 37 and 42 of the 53 member states (MSs), respectively, by 2018. Sixteen MSs are still endemic for measles, 11 for rubella and nine for both diseases, the latter including Italy. Elimination is documented by each country’s National Verification Committee (NVC) through an annual status update (ASU).

**Objective:**

By analysing data used to produce the ASUs, we aimed to describe the advances made by Italy towards elimination of measles and rubella. Moreover, we propose a set of major interventions that could facilitate the elimination process.

**Methods:**

A total of 28 indicators were identified within the six core sections of the ASU form and these were evaluated for the period 2013–2018. These indicators relate to the incidence of measles/rubella; epidemiological investigation of cases; investigation of outbreaks; performance of the surveillance system; population immunity levels; and implementation of supplemental immunization activities (SIAs).

**Results:**

From 2013 to 2018, epidemiological and laboratory analyses of measles cases in Italy improved substantially, allowing timely investigation in 2017 and 2018 of most outbreak and sporadic cases and identification of the majority of genotypic variants. Moreover, since 2017, vaccination coverage has increased significantly. Despite these improvements, several areas of concern emerged, prompting the following recommendations: i) improve outbreak monitoring; ii) strengthen the MoRoNet network; iii) increase the number of SIAs; iv) reinforce vaccination services; v) maintain regional monitoring; vi) design effective communication strategies; vii) foster the role of general practitioners and family paediatricians.

**Conclusions:**

The review of national ASUs is a crucial step to provide the NVC with useful insights into the elimination process and to guide the development of targeted interventions. Against this background, the seven recommendations proposed by the NVC have been shared with the Italian Ministry of Health and the Technical Advisory Group on measles and rubella elimination and have been incorporated into the new Italian Elimination Plan 2019–2023 as a technical aid to facilitate the achievement of disease elimination goals.

## Introduction

In the European Region of the World Health Organization (WHO), endemic transmission of measles and rubella has been interrupted by 37 and 42 out of 53 member states (MSs), respectively, by 2018. Sixteen MSs (30%) are still endemic for measles (of which four have re-established endemic measles transmission in 2018); 11 are still endemic for rubella and nine for both diseases, the latter including Italy [1].

In each MS, the National Verification Committee (NVC) is responsible to monitor and verify the elimination process at national level through the elaboration of an annual status update (ASU) which is annually submitted to the Regional Verification Commission (RVC) for review and evaluation [2]. Members of NVCs are designated by their respective ministers of health and include specialists from various fields who participate on a voluntary basis [2]. In Italy, the current NVC is in office since 2015 and has submitted a total of six ASUs to date (ASUs for 2013 and 2014 were both submitted in 2015). In addition, to verify elimination of measles and rubella also at the subnational level, regional reports and synthetic regional reports were produced for the years 2014–2016 within the project "Actions to support the National Plan for the Elimination of Measles and Congenital Rubella" [3–6].

Through an analysis of data used by the Italian NVC to write the national ASUs submitted for the years 2013–2018, this study aims to describe the progress made by Italy towards the elimination of measles and rubella, with a special focus on 2017 and 2018, and highlight the critical issues that are still hindering the achievement of such goals. We also recommend a set of major interventions that could improve the elimination process.

## Methods

The national ASU allows to verify the interruption of endemic measles and rubella transmission through five core components which relate to i) the epidemiology of measles, rubella and CRS cases (including incidence/cases; epidemiological investigation of measles/rubella cases; outbreak investigation); ii) the molecular epidemiology of measles and rubella viruses; iii) performance of measles, rubella, and CRS surveillance; iv) the population immunity against measles and rubella (which includes vaccination coverage and supplemental immunization activities (SIAs)); v) the sustainability of the national immunization programme (NIP) [2].

Data on the 1st core component are provided by the Department of Epidemiology of the National Institute of Health (Istituto Superiore di Sanità –ISS) based on information reported by the Regions using the web-based integrated measles and rubella surveillance platform. Data on the 2nd core component are provided by the National Reference Laboratory for Measles and Rubella (NRL) of the ISS, which, together with the Department of Epidemiology, provides also data on the performance of measles and rubella surveillance. Data on vaccination coverage are provided by the Italian Ministry of Health (MoH) based on the data collected by the Regions from the respective administrative territories; data on SIAs are provided directly by Regions, which are also required to provide information on the outbreak investigations conducted through the submission of the outbreak reporting forms. Information on activities and initiatives to support the NIP are generally provided by the MoH.

Within the ASU’s core components, a total of 28 indicators were identified (Table 1). Most of these indicators were used previously to monitor and evaluate measles elimination in Italy at subnational level for the period 2014–2016 [6].

**Table 1:** Number of indicators identified in each core component of the ASU’s form.

Component	Indicators (N)
*Epidemiology of measles*, *rubella and CRS cases*	6
*Molecular epidemiology of measles and rubella viruses*	0
*Performance of measles*, *rubella and CRS surveillance*	16
*Population immunity against measles and rubella*	6
*Sustainability of the national immunization programme*	0

The first three indicators are the standard indicators used by the WHO to assess the incidence of measles and rubella and the number of CRS cases. The 4th indicator relates to the number of outbreaks notified in a given year and has been newly created based on information contained in the outbreak section of the ASU. Moreover, since the WHO requires that for each outbreak being notified a report form, containing a description of the epidemic and the control and prophylaxis measures taken to contain its spread, should be completed by the regional surveillance systems [7], further two indicators have been created to assess the quality of outbreak investigation. Specifically, the 5th indicator relates to the percentage of outbreaks with submission of the outbreak reporting form, while the 6th indicator relates to the percentage of outbreak reporting forms that contain description of outbreak (e.g. index case, case contacts, setting, pattern of transmission) and measures taken. The 7th to 22nd indicators are WHO standard indicators used in the national ASU to assess the performance of measles and rubella surveillance systems [2]. The 23^rd^ to 26^th^ indicators are the standard vaccination coverage indicators for measles and rubella; the last two indicators, which have been drawn up from scratch, relate to the SIAs performed across the country (i.e. strategies for delivering vaccination to children missed by routine services or to older susceptible individuals) and the percentage of Regions reporting SIAs. WHO targets are available for standard indicators since these indicators are used by the RVC to make reliable conclusions regarding achievement of the elimination objectives in each MS.

The 28 indicators have been evaluated for the period 2013–2018. For those indicators for which WHO targets are not available, we evaluated only the national trends for the period considered; for indicators with available WHO targets, we also assessed the national data against the WHO targets (see Table 2 in the Results for details).

## Results

### Incidence of measles and rubella

Compared to previous years, a dramatic increase in the incidence of measles was observed in 2017, when measles incidence reached 88.4 cases per million population (5404 cases) (Table 2). In 2018, measles incidence decreased to 43.3 cases per million population, with 2682 measles cases reported to the Italian surveillance system. For rubella, despite a slight increase in 2017, the incidence remained relatively low over the period considered. Concerning CRS, no more than one case has been reported in Italy since 2014. In 2018, one imported case of CRS was reported to the surveillance system, but this was excluded from the total number of cases classified as CRS since, according to WHO guidelines, only endemic cases must be included in this count.

**Table 2:** Results of the analysis of data used in the national annual status updates, Italy 2013–2018.

Indicators	WHO targets	2013	2014	2015	2016	2017	2018
Incidence or number of cases							
1. *Measles incidence per 1 million population*	*<1/1*,*000*,*000*	40.7	27.0	3.9	13.6	88.4	43.3
2. *Rubella incidence per 1 million population*	*<1/1*,*000*,*000*	1.2	0.4	0.4	0.5	1.1	0.3
3. *Number of CRS cases*	*<1/100*,*000*	3	0	0	1	1	0
Outbreaks							
4. *Number of outbreaks*		203	204	38	119	634	231
5. *Percentage of outbreaks with submission of the outbreak reporting form*		0.0	89.7	84.2	57.1	100.0	100.0
6. *Percentage of outbreak reporting forms with information on description of outbreak and measures taken*		0.0	52.5	76.3	43.7	5.7	35.1
Performance of measles surveillance							
7. *Timeliness of reporting to national level (in %)*	*≥80%*	90.5	79.4	87.7	89.7	92.9	89.3
8. *Completeness of reporting to national level (in %)*	*≥80%*	95.2	79.4	87.7	89.7	92.9	89.3
9. *Rate of laboratory investigations (in %)*	*≥80%*	12.3	12.4	21.6	16.0	83.1	75.1
10. *Rate of discarded cases*	*≥2/100*,*000*	0.30	0.20	0.14	0.12	0.68	0.39
11. *Representativeness of reporting discarded cases (in %)*	*≥80%*	0.0	0.0	0.0	0.0	4.8	0.0
12. *Viral detection (in %)*	*≥80%*	20.5	NA	NA	32.5	62.1	71.9
13. *Origin of infection identified (in %)*	*≥80%*	93.3	95.7	98.4	93.5	90.9	89.2
14. *Timeliness of investigation (in %)*	*≥80%*	NA	NA	NA	NA	NA	0.0
Performance of rubella surveillance							
15. *Timeliness of reporting to national level (in %)*	*≥80%*	90.5	79.4	87.7	89.7	92.9	89.3
16. *Completeness of reporting to national level (in %)*	*≥80%*	90.5	79.4	87.7	89.7	92.9	89.3
17. *Rate of laboratory investigations (in %)*	*≥80%*	61.0	31.9	25.5	13.2	17.0	23.8
18. *Rate of discarded cases*	*≥2/100*,*000*	0.05	0.05	0.04	0.04	0.04	0.04
19. *Representativeness of reporting discarded cases (in %)*	*≥80%*	0.0	0.0	0.0	0.0	0.0	0.0
20. *Viral detection (in %)*	*≥80%*	NA	NA	NA	NA	NA	NA
21. *Origin of infection identified (in %)*	*≥80%*	68.2	76.0	66.7	83.3	83.8	61.9
22. *Timeliness of investigation (in %)*	*≥80%*	NA	NA	NA	NA	NA	NA
Routine vaccination coverage and SIAs							
23. *Measles-containing vaccine, 1st dose*	*≥95%*	90.35	86.74	85.29	87.26	91.68	92.99
24. *Measles-containing vaccine, 2nd dose*	*≥95%*	84.05	82.72	82.94	82.24	85.80	88.88
25. *Rubella-containing vaccine, 1st dose*	*≥95%*	90.30	86.69	85.22	87.19	91.64	92.97
26. *Rubella-containing vaccine, 2nd dose*	*≥95%*	83.65	82.46	82.75	82.04	85.62	88.73
27. *Number of SIAs*		5	14	44	34	15	58
28. *Percentage of Regions reporting SIAs*		14.3	28.6	38.1	38.1	14.3	42.9

*WHO* World Health Organization; *SIAs* supplemental immunization activities; *NA* not available.

### Outbreaks

The number of measles outbreaks in 2017 was 5-fold higher than the number registered in 2016 (634 vs 119); in 2018 a reduction was observed, with 231 outbreaks being reported to the national surveillance system (Table 2). It must be underlined that most of the outbreaks notified in the years under review consisted of only two cases (total number of outbreak cases between 2013 and 2018: 5007; median: 2; interquartile range: 2–3).

Unlike in previous years, both in 2017 and in 2018, Italy succeeded in submitting an outbreak reporting form for each outbreak identified. However, results for the indicator “percentage of outbreak reporting forms with information on description of outbreak and measures taken” were not satisfactory in most of the years under review, suggesting a weakness of the Italian surveillance system in monitoring the epidemiological investigation undertaken during outbreaks. The lowest result was reached in 2017, when only 5.7% of the outbreak forms contained this information.

### Surveillance performance

All except two performance indicators relating to measles surveillance improved in 2017 compared to previous years; the exceptions were the timeliness of investigation, currently not monitored for both measles and rubella, and the origin of infection identified which, albeit declining, was still above the WHO target of ≥80% (Table 2). A sharp increase was observed in particular in the rate of laboratory investigation, which increased from 16.0% in 2016 to 83.1% in 2017, thereby achieving the WHO target of ≥80%, and in the viral detection rate, which increased from 32.5% in 2016 to 62.1% in 2017. A similar improvement in performance indicators was observed in 2018, with the difference that the rate of laboratory investigation decreased by eight percentage points compared to 2017, while an increase of 9.8 percentage points on the previous year was observed for viral detection. Indicators relating to the performance of rubella surveillance did not improve to the same extent as those of measles. In particular, the rate of laboratory investigation has stagnated below 32% since 2014. The origin of infection identified achieved the WHO target of ≥80% in 2016 and in 2017; however, it significantly decreased in 2018, losing more than 20 percentage points compared to the previous two years. The indicator viral detection was not applicable since no rubella outbreaks occurred in the years under review.

### Vaccination coverage and SIAs

Vaccination coverage has begun to increase substantially since 2017. The most recent data available show that vaccination coverage for measles-containing vaccine (MCV) increased by 5.7 percentage points in 2018 compared to 2016 with regard to the 1^st^ dose (87.3% in 2016 vs 93.0% in 2018) and by 6.6 percentage points with regard to the 2^nd^ dose (82.2% in 2016 vs 88.9% in 2018). Almost identical trends were observed for the first and second doses of rubella-containing vaccine (RCV; Table 2).

The implementation of SIAs increased substantially in the first three years under review, with 44 SIAs reported in 2015 vs 5 SIAs in 2013. The number of Regions reporting SIAs also increased over this period (14.1% vs 38.1%). However, in 2016, a decrease in the number of SIAs was observed, which became more severe in 2017; the number of Regions reporting SIAs also decreased dramatically in 2017. In contrast, in 2018 a marked improvement in both these indicators was observed, with nine Regions out of 21 (42.9%) reporting an overall number of 58 SIAs (Tables 2 and 3).

**Table 3:** Frequency of SIAs and of regions reporting SIAs by target population, Italy, 2013–2018.

Year	2013		2014		2015		2016		2017		2018	
Total number of SIAs	5		14		44		34		15		58	
Regions reporting SIAs	3		6		8		8		3		9	
Target population	SIAs	Regions	SIAs	Regions	SIAs	Regions	SIAs	Regions	SIAs	Regions	SIAs	Regions
	N (%)	N	N (%)	N	N (%)	N	N (%)	N	N (%)	N	N (%)	N
*All ages*	1 (20)	1	1 (7.1)	1	1 (2.3)	1	−	−	−	−	1 (1.7)	1
*Children (0–10 years)*	−	−	6 (42.9)	3	12 (27.3)	4	12 (35.3)	6	10 (66.7)	2	31 (53.4)	6
*Adolescents (11–18 years)*	1 (20)	1	7 (35.7)	4	21 (47.7)	5	13 (38.2)	5	5 (33.3)	1	24 (42.9)	7
*Adults (19–64 years)*	−	−	−	−	7 (15.9)	3	1 (2.9)	1	1 (6.7)	1	1 (1.7)	1
*Women of child-bearing age/Post-partum women*	3 (60)	2	3 (21.4)	2	3 (21.4)	3	4 (11.8)	2	−	−	−	−
*Medical or HealthCare Workers/students*	−	−	1 (7.1)	1	1 (2.3)	1	1 (2.9)	1	−	−	2 (3.4)	2
*Migrants/refugees*	−	−	1 (7.1)	1	2 (4.5)	2	3 (8.8)	3	1 (6.7)	1	5 (8.6)	4
*Roma population camps*	−	−	1 (7.1)	1	1 (2.3)	1	3 (8.8)	1	−	−	4 (6.9)	3
*Detainees*	−	−	−	−	−	−	−	−	−	−	1 (1.7)	1

Note: Frequency of SIAs/Regions reporting SIAs by target population may differ from the total number of SIAs/Regions reporting SIAs in Table 2 since some SIAs were targeted to more than one population group.

Globally, over 90% of the reported SIAs (80% in 2013; 92.9% in 2014; 97.7 in 2015; 91.2% in 2016; 93.3 in 2017; 98.3 in 2018) were aimed at increasing population immunity through the monitoring of non-compliant or susceptible subjects and the active vaccination offer to broad age groups or high-risk population groups. The remaining portion has been finalized to the interruption of the epidemic chain through active case search and vaccination offer to all susceptible contacts.

During the period 2013–2018, the age groups most represented in the SIAs were children (0–10 years) and adolescents (11–18 years) with percentages, out of the total number of SIAs, that reached 67% for children in 2017 and 48% for adolescents in 2015 (Table 3). In the years 2013–2016, 28.7% of SIAs were addressed to women of childbearing age/post-partum period; this percentage has dropped dramatically to zero in the last two years. Since 2014, the trend over time of SIAs dedicated to migrants/refugees seeking-asylum (7.1% in 2014, 8.6 in 2018) or to the Roma population (7.1% in 2014, 6.9% in 2018) appears to be quite stable (Table 3).

### Regional differences

In countries with decentralized health policies, progress towards elimination should also be assessed at the subnational level [2, 6]. In Italy, which consists of 21 administrative Regions, regional differences can hinder the achievement of elimination goals [6]. This is particularly evident for vaccination coverage, which is a key measure for evaluating the performance of the immunization system at both national and subnational level [2].

**Fig 1:**
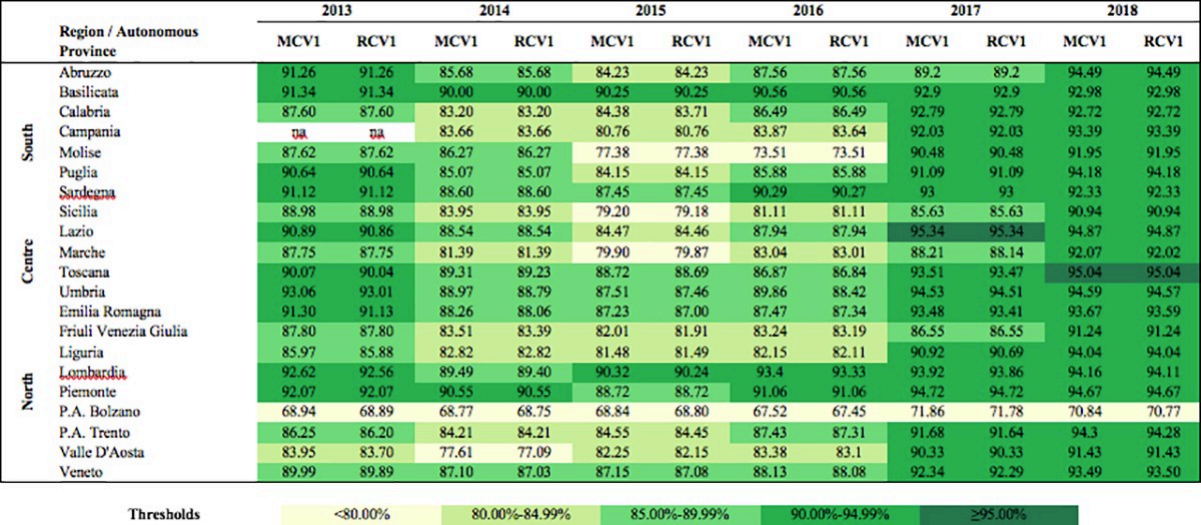
Vaccination coverage for measles- and rubella-containing vaccine first dose by Region, Italy, 2013–2018. *MCV1*, measles-containing vaccine first dose; *RCV1*, rubella-containing vaccine first dose.

**Fig 2:**
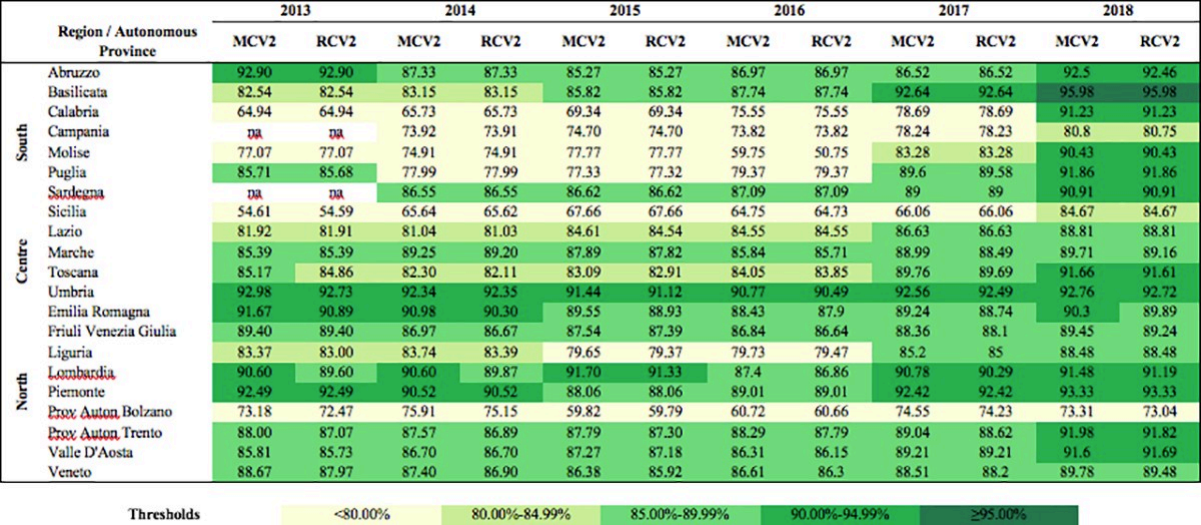
Vaccination coverage for measles and rubella-containing vaccine second dose by Region, Italy, 2013–2018. *MCV2*, measles-containing vaccine second dose; *RCV2*, rubella-containing vaccine second dose.

Figs 1 and 2 show regional vaccination coverage for the period 2013–2018, for the first and second doses, respectively, of MCV and RCV. These are the official data published by the Italian Ministry of Health and may differ from the ASU data due to the fact that the former are generally released after the ASU submission and can therefore be more uptodate. Most of the Regions of the South registered rates below the national average until 2016; this was particularly evident for the second dose (Fig 2). Regional differences between 2014 and 2016 have been amply described elsewhere [6]. In 2017 and in 2018 a greater homogeneity among both Regions and geographical areas was achieved for the first dose, with 16 and 20 Regions achieving a vaccination coverage higher than 90%, respectively (Fig 1). In contrast, heterogeneity remained high for the second dose in both years (Fig 2). In 2018, only two Regions reached the 95% target, Toscana for the first dose and Basilicata for the second dose.

## Discussion

Between 2013 and 2018, there were two clear advancements towards the elimination of measles and rubella in Italy. Laboratory investigation and molecular epidemiology of measles cases are undoubtedly an area in which considerable improvements have been achieved. Such improvements can mostly be attributed to the establishment of the National Network of Reference Laboratories for measles and rubella (MoRoNet), supervised by the NRL [8]. The work carried out by this network enabled a timely laboratory investigation of the outbreak and sporadic cases that occurred in 2017, and the identification of the majority of the genotypic variants circulating in the country, as well as confirmation of episodes of disease imported from abroad [9]. The performance of measles laboratory surveillance also continued to be good in 2018. Despite a decrease in the rate of measles laboratory investigations (83.1% in 2017 vs. 75.1% in 2018)–which can be explained by a poorer performance in some Italian Regions, probably due to a reduction in the efficiency of local health systems–detection of measles virus from outbreaks further improved in 2018, reaching 71.9% (from 62.1% in 2017). Molecular epidemiology of measles viruses is indeed an important component of outbreak investigation and is essential for global surveillance of circulating wild–type measles strains. Genetic analyses performed by the NRL on measles virus sequences coming from the network’s laboratoriesshowed in particular the co-circulation of genotypes B3 and D8 in 2018. The B3-Dublin, B3-Saint Denis and D8-GirSomnath were the most prevalent named strain variants within genotype B3 and D8. Others named strain variants were less frequently identified (this topic will be treated in more detail in a specific paper).

Another positive aspect that must be underlined is the increase in measles and rubella vaccination coverage registered in the last two years of the study: 5.7 percentage points more for the first dose and 6.6 percentage points more for the second dose compared to 2016. This progress is the result of a marked increase in the political commitment of Italy to measles and rubella elimination. A major example of this increased commitment is the law n° 119/2017, which was approved in Italy in 2017 with the aim of making ten vaccinations, including measles and rubella, mandatory for children from birth to 16 years of age [10]. Together with Italy, other European countries enforced compulsory policies in order to prevent the spread of infectious diseases. With specific regard to measles and rubella, a total of nine countries (Bulgaria, Croatia, Czech Republic, France, Hungary, Italy, Latvia, Poland, and Slovakia) adopted mandatory vaccination against mumps-measles-rubella, while this vaccination is recommended in the other 22 European countries [11]. Other examples of the increased commitment of Italy to measles and rubella elimination are the approval of the new National Plan of Vaccine Prevention (PNPV) 2017–2019, which became officially operative in January 2017 and confirms the elimination of measles and rubella as a priority national objective [12], and the 2017 Financial Law, which establishes a specific fund for the supply of vaccines by the Regions [13].

However, despite these improvements, the elimination of measles and rubella in Italy is not yet on the horizon. The analysis of the ASUs revealed in fact some critical aspects of the Italian elimination programme that still need to be addressed. One of these aspects is the poor quality of outbreak monitoring highlighted by the low number of report forms submitted and the incomplete nature of the relevant information. There are two reasons for this. Firstly, the great number of outbreaks with only two cases might have discouraged the conduction of thorough outbreak investigations. Secondly, to date, Regions produce such report forms only in retrospect in response to a specific ministerial request, with the consequence that some information can be lost.

Another critical aspect is the performance of rubella surveillance for which no improvements were observed in 2017 and in 2018, especially with regard to the laboratory investigation and the origin of infection identified. One possible explanation is that rubella is generally a milder disease than measles and infection is subclinical in 30%–50% of cases; therefore, although measles and rubella surveillance are integrated, surveillance is less sensitive for rubella [14]. This situation is not limited only to Italy. In fact, based on the evaluations made by the RVC on the ASUs submitted by MSs for 2018, the extent and quality of rubella surveillance is still suboptimal in many countries [1].

Finally, SIAs have been poorly implemented in the years under review. This situation already emerged from the monitoring conducted at the regional level for the years 2014–2016 that showed that over half of the Regions did not report a single SIA for the period considered [6]. The situation worsened further in 2017, when only three Regions out of 21 (14.3%) reported SIAs, giving a total of just 15 immunization activities across the whole country. In 2018, although an improvement in the number of SIAs was observed, the proportion of reporting Regions was still below 50%. With regard to groups targeted by SIAs, the need to rapidly increase vaccination coverage in the light of the high number of measles cases of recent years has probably led Regions to conduct SIAs primarily targeting children and adolescents. On the contrary, less attention has been paid to the older age groups. In fact, only two SIAs out of the 73 conducted in 2017 and in 2018 were addressed to adults not belonging to specific risk groups. Similarly, very few SIAs were implemented to increase vaccination coverage of at-higher-risk groups such as health care workers (HCWs), targeted, on average, by only one SIA per year, and women of childbearing age, for which no SIAs were reported in the last two years under review.

Several issues are still hindering the achievement of the elimination goals in our country. In view of these problems, a set of major interventions aimed at improving the elimination process have been recommended by the Italian NVC.

### Improve outbreak monitoring

To increase vaccination services capacity to report outbreak investigations, outbreak report forms should be completed directly on the web-based surveillance platform. This would not only avoid data loss due to an ex post analysis, but would allow, on a central level, timely monitoring of epidemics and the implementation of targeted containment actions. Based on this recommendation of the NVC, a ministerial circular was released in November 2018 to further strengthen the national integrated measles and rubella surveillance system [15], including among the main changes the integration of the outbreak report form in the surveillance platform to improve collection of relevant outbreak information (e.g. source of infection, transmission settings, vaccination details). The update will also include the possibility of specifying the date of initiation of the epidemiological investigation, thus enabling the calculation of the indicator “Timeliness of investigation”, which is not currently monitored.

### Strengthen the MoRoNet network

The MoRoNet network has to be further strengthened to ensure that the quality of rubella laboratory data meet the WHO standard requirements along with those of measles. In this respect, in November 2018, the Italian Ministry of Health released a circular reporting indications related to laboratory diagnosis. The document underlines the importance of ensuring that measles and rubella diagnosis and genotyping is performed by an accredited laboratory belonging to MoRoNet.

### Increase SIAs

Although the recent legislation on mandatory vaccination should lead to an improvement in vaccination coverage for individuals up to the age of 16 years, additional immunization activities are needed to increase vaccination coverage rates in older age groups, particularly given the high percentage of cases in the adult population. Major efforts are also needed to improve vaccination coverage of at-higher-risk groups such as HCWs. In Italy, HCWs are not required to show evidence of measles immunity for employment. Verifying the immunity status of all HCWs and ensuring that susceptible HCWs are vaccinated against measles is therefore of paramount importance [16–18]. Greater attention should also be given to women of childbearing age both for the elimination of congenital rubella and for the protection of the newborn before vaccination and, more generally, because they represent a fundamental target for the promotion of vaccination culture and for contrasting vaccine hesitancy [19–21]

### Reinforce vaccination services

The introduction of the law on mandatory vaccination has led to an increased commitment to routine immunization activities among regional vaccine services. However, this might be at the expense of other activities, such as routine epidemiological investigations and SIAs, to which, especially in 2017, less time and fewer resources have been devoted in an effort to enact the measures provided for by the new legislation. To allow vaccination services to carry out both routine, high-quality epidemiological investigations and SIAs, it is essential to increase their financial support as well as their workforce.

### Maintain the regional monitoring

The Regions use different organizational models for the delivery of vaccination services, which has meant that, even with the new law, the increase in routine vaccination coverage observed in 2017 and in 2018 was not homogeneous across the country, especially for the second dose of both measles and rubella vaccine. This situation was already apparent from the analysis of regional vaccination coverage conducted for the period 2014–2016 [6], which showed poor results in Regions with financial deficit, although these poor results were not confined to vaccination. Decentralization policies have certainly led to significant differences among regional health services in the whole area of health promotion and disease prevention [22–26]. Thus, monitoring progress towards the elimination goals at subnational as well as national level appears to be essential to ensure the uniform implementation of vaccination policies across the country. The local monitoring carried out through the regional ASUs and the SSRs has proved to be very useful for this purpose. It has highlighted the most critical areas in the elimination programme at regional level and has identified those Regions experiencing the most difficulty in achieving the objectives of the programme, resulting in the development of plans for targeted activities to support these Regions [3–6, 27]. The production of such reports should therefore continue to complement that of the national ASU to guarantee a fine-scale monitoring of the elimination programme.

### Design effective communication strategies

Vaccine hesitancy has been increasing worldwide and in particular in European countries, including Italy, where it has led to a decline in vaccination coverage for several vaccines, resulting in continued outbreaks of vaccine-preventable diseases such as measles [28]. In Italy, to counteract the growing scepticism towards vaccination, the Italian parliament approved law no. 73/2017 in June 2017. This legislative measure, however, became the subject of great debate and many conflicting opinions. In response to this debate, the Five Star Movement–League government has proposed a new bill introducing a so-called “flexible obligation”, which would prioritize the education of parents on the benefits of vaccines without making immunization compulsory, unless there are disease outbreaks or the vaccination coverage rate falls too low [29]. The debate is still ongoing, but, according to Ministry of Health press releases, measles and rubella vaccinations will remain mandatory. Within this context, it is clear that legislative measures alone are not sufficient to guarantee that elimination goals are achieved and that initiatives aimed at increasing the trust of Italian citizens in vaccination are urgently needed. In particular, there is the need to reinforce parents’ beliefs about the importance of vaccination and to increase their knowledge regarding the benefit of vaccines. Parental knowledge of and attitudes towards the efficacy and safety of vaccines may have indeed an effect on parents’ decision of vaccinating their children [30, 31]. To this end, it is essential to train HCWs involved in administering vaccines by providing them with technical and evidence-based information on vaccines in order to ensure that they can communicate effectively with parents [32]. Furthermore, it is important to encourage greater activism of institutions and scientific societies on the web and social media to counteract disinformation precisely where fake news on vaccines is mainly disseminated and to provide parents with reliable information on the validity and benefit of vaccination [33–36].

### Foster the role of general practitioners and family paediatricians

General practitioners and family paediatricians are a reliable source of information for parents with children due for vaccination. A national survey on parental vaccine hesitancy in Italy revealed that over 90% of respondents consider their family paediatrician to be a very or sufficiently reliable source of information on vaccinations [37]; in another Italian survey investigating knowledge and attitudes on paediatric vaccinations in pregnant women, almost 80% of those that obtained information on vaccination from general practitioners or primary care paediatricians indicated that the quality of the information provided was satisfactory [21].

However, an extensive critical review has shown that the quality of the information itself seems to matter less than the credibility of the institutions that deliver it [38]. Health care professionals should therefore be adequately trained to improve not only their technical but also their communication skills to ensure that unambiguous messages are delivered to patients and parents, to build trust in vaccination throughout the population.

## Conclusions

Besides promoting the documentation and verification process, the NVC advocates for strengthening measles and rubella elimination programme by encouraging authorities to implement appropriate strategies towards elimination goals. The review of national ASUs is a crucial step to provide the NVC with useful insights into the elimination process and to guide the development of targeted interventions. Against this background, the seven recommendations proposed by the NVC have been shared with the Italian Ministry of Health and the National Technical advisory group as a technical aid to support the elimination process. Moreover, such recommendations have been incorporated into the draft of the new Italian Measles and Rubella Elimination Plan 2019–2023, which is currently being evaluated by the State-Regions Conference and will likely be released as soon as an agreement between State and Regions is reached.
